# Progressive hemi facial atrophy - Parry Romberg syndrome presenting as severe facial pain in a young man: a case report

**DOI:** 10.4076/1757-1626-2-6776

**Published:** 2009-07-02

**Authors:** Anita A Kumar, Rajesh A Kumar, Ghanshyam Palamaner Subash Shantha, Ghanshyam Aloogopinathan

**Affiliations:** Department of General Medicine, Sri Ramachandra UniversityPorur, Chennai-600116, Tamil NaduIndia

## Abstract

We present a 30-year-old South Indian man who presented with complaints of left sided headache and facial pain for past 3 months, severe for past 10 days. On physical examination, right side of the face appeared normal. Left side of the face showed signs of hemi atrophy with minimal drooping of left eyelid. All Systems were found to be normal. Routine blood and urine investigations results were within normal limits. X-ray chest revealed no abnormalities and x-ray skull showed both sides equal. Computerized tomogram of the brain showed left minimal sub dural hygroma with no midline shift, and no evidence of cerebral edema or cerebral atrophy. Nerve conduction study showed features suggestive of trigeminal neuralgia. MRI of the skull base was also normal and showed no evidence of trigeminal nerve compression. Interestingly, he had minimal response to analgesics, steroids, and propranolol, but showed immediate response to carbamazepine. Hence this patient indeed had Parry Romberg syndrome: Hemi facial atrophy with trigeminal neuralgia.

## Introduction

Unilateral progressive atrophy of the face was first described by Parry in 1825 and by Romberg in 1846. Eulenberg coined the term ‘progressive facial hemi atrophy’ in 1871. There is involvement of the skin and subcutaneous fat, and on rare occasions also of the muscles and bones. It is uncommon and generally unilateral with a higher incidence rate in females. The extension of the atrophy is frequently limited to one side of the face, and the ipsilateral involvement of body is rare. Ocular involvement is common, and the most frequent manifestation is enophthalmy. The etiology of the disease is unidentified. Occasionally, there may be some neurological complications, such as trigeminal neuralgia, facial paresthesia, severe headache and contra lateral epilepsy. Patients, who manifest atrophy in early ages, have a better outcome. Here we report one such patient with Parry Romberg syndrome who presented with trigeminal neuralgia.

## Case presentation

**Figure 1.**
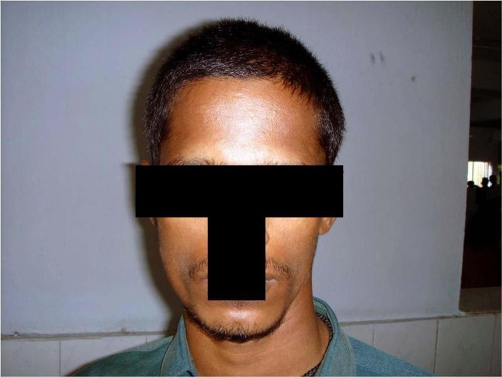
Patient showing unilateral hemi facial atrophy. Photograph of the Patient’s face showing facial atrophy to the left side.

**Figure 2.**
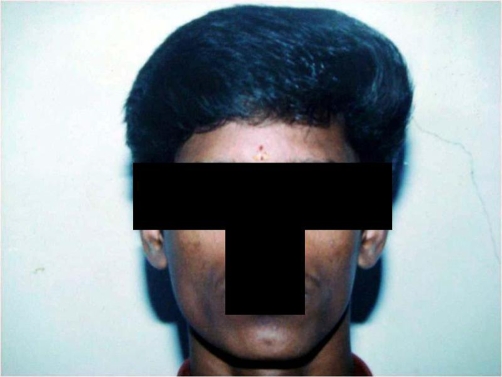
Patient showing no obvious abnormality 5 years ago. Photograph of the patient’s face showing symmetry of both sides.

**Figure 3.**
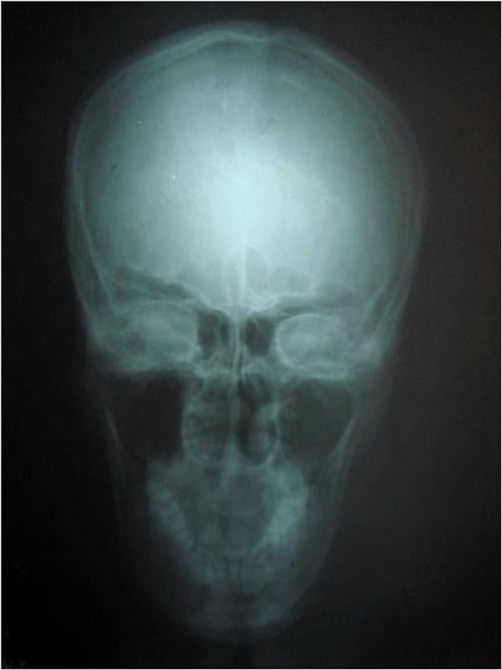
Photograph of the X-ray skull showing both sides equal.

**Figure 4.**
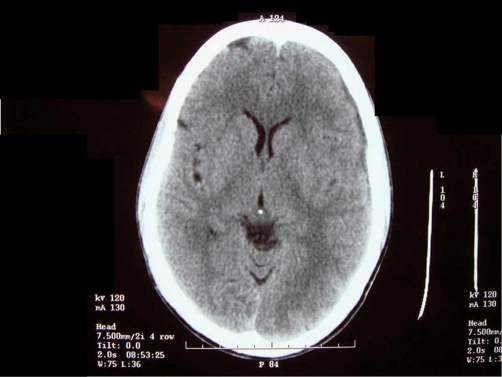
Photograph of the CT brain showing left minimal sub dural hygroma with no midline shift and no evidence of cerebral edema or cerebral atrophy.

A 30-year-old South Indian man presented with complaints of left sided headache and left sided facial pain, which was intermittent, sharp stabbing quality for past 3 months, severe for past 10 days. He had no history of fever, convulsions, loss of consciousness or ear, nose, throat bleed. Patient gave past history of head injury 10 years ago to the left skull for which he took no treatment and no imaging studies were done. Not a known diabetic or hypertensive. Patient denies history of smoking or alcohol intake. He is married with one child. Patient was moderately built and nourished with a body mass index (BMI) of 21 kg/m2. On physical examination, he was conscious, oriented, afebrile, general condition was fair and vitals stable. Right side of the face appeared normal. Left side of the face showed signs of hemi atrophy with minimal drooping of left eyelid (Figure 1). Examination of the face revealed no sensory or motor deficits on both sides. Further, there was no other evidence of Horner’s syndrome, facial palsy, or hemi facial spasm. 5 years ago the same patient appeared normal with no obvious facial abnormality (Figure 2). Systemic examination of the central nervous system (CNS) was normal. Fundus examination was normal. All other systems were found to be normal. Local examination, measurement were taken from the nasion to the tragus, nasion to angle of mandible, and mid chin to tragus of both right and left side (Table.1). The measurements showed hemi facial atrophy of the left side. Routine blood and urine investigations showed within normal limits (Table 2). Diagnostic radiological imaging were done in which ultrasound abdomen (USG abdomen) showed no organomegaly or free fluid. X-ray chest revealed no abnormalities and X-ray skull showed both sides equal (Figure 3). Computerized tomogram brain (CT) showed Left minimal sub dural hygroma with no midline shift, and no evidence of cerebral edema or cerebral atrophy (Figure 4). Nerve conduction study showed features suggestive of trigeminal neuralgia of left side. In view of the persistent headache, a neurologist’s opinion was sought and concluded that the subdural hygroma, which was very minimal, was not the cause for the headache. Supportive and conservative management showed satisfactory response. Interestingly, he had minimal response to analgesics, steroids, and propranolol, but showed immediate response to carbamazepine. Carbamezipine 200 mg three times a day was initiated. After just 2 days he showed remarkable improvement. This treatment was continued for 1 month and then gradually tapered and stopped. At the end of treatment, there were no symptoms of neuralgia, though facial hemiatrophy persisted. Patient is under regular follow-up for 1 year and has not shown any recurrence.

**Table 1.** Initial Investigations

Diameters	Right side	Left side
Nasion to tragus	15 cms	13.3 cms
Nasion to angle of mandible	13 cms	11.5 cms
Mid Chin to tragus	16 cms	14.1 cms

## Discussion

Unilateral progressive atrophy of the face was first described by Parry in 1825 and Romberg in 1846. Eulenberg coined the term ‘progressive facial hemi atrophy’ in 1871. The term progressive hemi facial atrophy (PHA) is more widely accepted [1]. The disease manifests in the first or second decade of life with a slow progression over many years showing atrophy and then becomes stable [2-5]. Alterations concerning involvement, duration and deformity can stabilize in any stage of growth and development [2,6]. There is involvement of the skin and subcutaneous fat, and on rare occasions also of the muscles and bones [1]. Although, it is uncommon and generally unilateral [6,7], 5% to 10% of cases were described as being bilateral [6]. The extension of the atrophy is frequently limited to one side of the face, and the ipsilateral involvement of body is rare. Ocular involvement is common, and the most frequent manifestation is enophthalmy, due to fat loss around the orbit. The eye usually works normally and the ears can be smaller than normal ones, due to the atrophy [2]. Parry Romberg syndrome is found to be more common in females [7-9]. The etiology of the disease is unidentified. A cerebral disturbance on fat metabolism has been proposed as a primary cause [8,10,11]. Trauma, viral infections, endocrine disturbances, auto-immunity and heredity are believed to be also associated to the pathogenesis of the disease [2,9,10,12,13]. Occasionally, there may be some neurological complications, such as trigeminal neuralgia, facial paresthesia, severe headache and contra lateral epilepsy [4,6,14-17]. Contra lateral epilepsy is the most common complication as reported by Chbicheb M et al [17]. Parry-Romberg Syndrome is a self-limiting condition and there is no cure. Patients, who manifest atrophy in early ages, have a better outcome [4]. Affected patients should have multidisciplinary attendance of physicians, dentists, phonoaudiologists and psychologists. Careful diagnosis is relevant, mainly in cases of systemic sicknesses with unknown origin, in which a simple anamnesis and a conventional clinical exam do not give enough data for a precise diagnosis and an appropriate treatment. Since our patient had hemi facial atrophy and neuralgic pain and dramatic response to carbamazepine, we made the diagnosis of Parry Romberg Syndrome. Since he came for pain we treated his symptom. He did not worry about his facial atrophy and hence no reconstructive surgery was performed.

## Conclusion

In conclusion, we present a case of left sided neuralgic pain with progressive hemi facial atrophy. All investigations showed within normal limits. Diagnostic imaging showed no anatomical defect relating to the patients symptoms. Hence this is indeed a rare case of Parry Romberg Syndrome with trigeminal neuralgia.

## Abbreviations

BMI: Body mass index
CNS: central nervous system
CT: Computerized tomogram
ENT: Ear, nose, throat
LOC: Loss of consciousness
PHA: progressive hemi facial atrophy
USG abd: ultrasound abdomen

## References

[bib-001] Goldhammer Y, Kronenberg J, Tadmor R, Braham J, Leventon G (1981). Progressive hemifacial atrophy (Parry-Romberg's disease), principally involving bone. J Laryngol Otol.

[bib-002] Mazzeo N, Fisher JG, Mayer MH, Mathieu GP, Mcade FGG (1995). Progressive hemifacial atrophy (Parry Romberg Syndrome). Oral Surg Oral Med Oral Pathol Oral Radiol Endod.

[bib-003] Moore MH, Wong KS, Proudman TW, David DJ (1993). Progressive hemifacial atrophy (Romberg´s Disease): skeletal involvement and treatment. Br J Plast Surg.

[bib-004] Neville BW, Damm DD, Allen CN, Bouqout JE, Guanabara Koogan (1998). Facilio Sugerica: Patologia oral e Maxilofacial.

[bib-005] Roddi R, Riggio E, Gilbert PM, Houvius SER, Vaandrager JM, van der Meulen JCH (1994). Clinical evaluation of techiniques used in the surgical treatment of progressive hemifacial atrophy. J Craniomaxilofac Surg.

[bib-006] Lakhani PK, David TJ (1984). Progressive hemifacial atrophy with scleroderma and ipsilateral limb wasting (Parry Romberg Syndrome). J R Soc Med.

[bib-007] Jurkiewicz MJ, Nahai F (1985). The use of free revascularized grafts in the amelioration of hemifacial atrophy. Plast Reconstr Surg.

[bib-008] Finesilver B, Rosow HN (1938). Total hemiatrophy. JAMA.

[bib-009] Pensler JM, Murphy GF, Muliken JB (1990). Clinical and ultra-structural studies of Romberg´s hemifacial atrophy. Plast Reconstr Surg.

[bib-010] Miller MT, Sloane H, Goldberg MF, Grisolano J, Frenkel M, Mafee MF (1987). Progressive hemifacial atrophy (Parry Romberg Disease). J Pediatr Oftamol Strabismus.

[bib-011] Foster TD (1979). The effects of hemifacial atrophy of dental growth. Br Dent J.

[bib-012] Shaffer WG, Hine MK, Levy BM, John S (1983). Hemifacial atrophy. Textbook of oral pathology.

[bib-013] Zafarulla MY (1985). Progressive hemifacial atrophy: a case report. Br J Ophthalmol.

[bib-014] Gorlin RJ, Pinborg JJ, Randman R (1964). Parry Romberg syndrome. Syndromes of the head and neck.

[bib-015] Roed-Petersen B (1979). Hemifacial lipodystrophy - report of a case. Oral Surg Oral Med Oral Pathol.

[bib-016] Sagild JC, Alving J (1985). Hemiplegic migraine and progressive hemifacial atrophy. Ann Neurol.

[bib-017] Chbicheb M, Gelot A, Rivier F, Roubertie A, HumbertClaude V, Coubes P, Echenne B (2005). Parry-Romberg´s syndrome and epilepsy. Rev Neurol.

